# Calcium levels modulate embryo yield in *Brassica napus* microspore embryogenesis

**DOI:** 10.3389/fpls.2024.1512500

**Published:** 2025-01-16

**Authors:** Antonio Calabuig-Serna, Ricardo Mir, Daniel Sancho-Oviedo, Paloma Arjona-Mudarra, Jose M. Seguí-Simarro

**Affiliations:** Cell Biology Group - Instituto para la Conservación y Mejora de la Agrodiversidad Valenciana (COMAV) Institute, Universitat Politècnica de València, Valencia, Spain

**Keywords:** androgenesis, Ca^2+^, *in vitro* culture, *in vitro* embryogenesis, morphogenesis, doubled haploids

## Abstract

Calcium (Ca^2+^) is a universal signaling cation with a prominent role as second messenger in many different plant processes, including sexual reproduction. However, there is much less knowledge about the involvement of Ca^2+^ during *in vitro* embryogenesis processes. In this work we performed a study of Ca^2+^ levels during the different stages of microspore embryogenesis in *Brassica napus*, with special attention to how Ca^2+^ can influence the occurrence of different embryogenic structures with different embryogenic potential. We also performed a pharmacological study to modulate Ca^2+^ homeostasis during different stages of the process, using a series of Ca^2+^-altering chemicals (BAPTA-AM, bepridil, chlorpromazine, cyclopiazonic acid, EGTA, inositol 1,4,5-trisphosphate, ionophore A23187, W-7). This study shows that Ca^2+^ increase can be considered as an early marker of induction of microspore embryogenesis. Besides, Ca^2+^ levels are highly dynamic during microspore embryogenesis, influencing the final embryo yield. Increase of either extracellular or intracellular Ca^2+^ levels improves embryo yield without altering the proportion of highly embryogenic structures formed, which suggests that elevated Ca^2+^ levels increase the amount of microspores reaching the minimum Ca^2+^ threshold required for embryogenesis induction. Conversely, inhibition of Ca^2+^ uptake or signaling results in reduced embryogenic response. This allows to modulate embryo yield within a functional range, with lower and upper Ca^2+^ thresholds beyond which embryo yield is reduced. There seems to be a relationship between Ca^2+^ levels and embryo differentiation.

## Introduction

Hybrid seed, which is by far the most used worldwide, is produced by crossing two homozygous (pure) parental lines. Pure lines can be generated by multiple self-crossing generations, which may last up to 7-10 years depending on the species and the desired homozygosity degree. Alternatively, *in vitro* culture of immature gametophytes produces haploid embryos that, either naturally or in an induced manner, can develop doubled haploid (DH), fully homozygous individuals in a single generation (Seguí-Simarro et al., 2021b). To date, protocols to produce haploids or DHs have been reported for nearly 400 species (Seguí-Simarro et al., 2021a). One of the *in vitro*-induced pathways for DH production is microspore embryogenesis, whereby vacuolated microspores or young pollen grains deviate from their natural gametophytic fate towards embryogenesis (Seguí-Simarro, 2010). Factors such as the genotype of donor plants are key to determine the embryogenic response of microspores. Whereas some species such as *Brassica napus* or tobacco are highly embryogenic, the response of other species such as eggplant or pepper is still limited, or even null as in tomato (Seguí-Simarro and Nuez, 2005; Corral-Martínez and Seguí-Simarro, 2012, 2014; Parra-Vega et al., 2013; Seguí-Simarro, 2016; Rivas-Sendra et al., 2020; Mir et al., 2021). Even within the same species, there are enormous differences between genotypes, as is the case in *B. napus* for the highly responding DH4079 line and the low-responsive DH12075 line (Camacho-Fernández et al., 2021, 2024; Corral-Martínez et al., 2021). The developmental stage of isolated microspores/pollen, the *in vitro* culture conditions, including the type of inductive treatment applied and the composition of the culture medium, are also key factors (Seguí-Simarro and Nuez, 2008; Seguí-Simarro, 2010; Seguí-Simarro et al., 2021b). As seen, there are many different intervening factors whose elucidation would help to improve the efficiency of the process, principally in recalcitrant backgrounds. However, the nature of the triggering signal that transform microspores into embryos remains elusive.

Aside of its structural role in the cell wall, forming a pectate gel with pectin (Micheli, 2001), calcium in its cationic form (Ca^2+^) is a fast and universal second messenger in multiple plant processes, including stress response, cell division and growth, pollen development, and embryogenesis and establishment of embryo polarity (Hause et al., 1994; Tian et al., 2020). Signaling is mediated by binding principally to calmodulin (CaM), a Ca^2+^-dependent protein that regulates the activity of a number of enzymes, ion channels, and other proteins with many diverse roles in cell function. Ca^2+^ signaling is involved on the induction of *in vitro* somatic embryogenesis (Overvoorde and Grimes, 1994; Mahalakshmi et al., 2007; Calabuig-Serna et al., 2023c). Indeed, addition of Ca^2+^ to the induction medium enhances somatic embryo yield (Jansen et al., 1990; Ramakrishna et al., 2012; Calabuig-Serna et al., 2023a). As to microspore embryogenesis, induction in most species involves the application of a heat stress. In general, the first perception of heat stress occurs through changes in plasma membrane fluidity which, together with the activation of stress-specific Ca^2+^ permeable channels, causes a transient increase in cytoplasmic Ca^2+^ levels. This, in turn, leads to increased Ca^2+^-CaM binding and the expression of several heat shock (HS) genes (Liu et al., 2005). In wheat, external Ca^2+^ is required for embryogenic commitment, a process where Ca^2+^ plays a role in signal transduction, since both reduced Ca^2+^ concentrations in the medium and CaM inhibition suppressed embryogenesis induction (Reynolds, 2000). Similarly, Ca^2+^ was associated to enhanced induction frequency and improved embryo structure in *Solanum carolinense* (Reynolds, 1990), *Hordeum vulgare* (Hoekstra et al., 1997) or *Triticum aestivum* (Cho and Kasha, 1995).

Traditionally, the dynamics of Ca^2+^ levels in plant embryogenesis has been studied using three principal approaches: Ca^2+^ modulators to alter Ca^2+^ levels, CaM-interacting chemicals to interfere with Ca^2+^ binding to CaM, and Ca^2+^ probes and sensors to track changes in Ca^2+^ levels. For example, to study the role of Ca^2+^ during somatic embryogenesis, the ionophore A23187 has been used to increase the permeability of the plasma membrane to Ca^2+^, BAPTA and EGTA (or their derivatives) for Ca^2+^ chelation, or W-7 as a CaM antagonist (Jansen et al., 1990; Overvoorde and Grimes, 1994; Takeda et al., 2003; Rivera-Solís et al., 2018; Calabuig-Serna et al., 2023b, 2023). Ca^2+^ sensors such as the genetically-encoded *cameleon* construct are FRET-based tools very convenient for the detection of small and transient Ca^2+^ changes (Krebs et al., 2012), and have been previously used to detect calcium dynamics during somatic embryogenesis in Arabidopsis and carrot (Krebs et al., 2012; Calabuig-Serna et al., 2023a, c). However, this technology relies on the availability of efficient protocols for genetic transformation, which is not the case for the DH4079 *B. napus* line (Calabuig-Serna et al., 2023b). Regarding Ca^2+^ probes, Ca^2+^-binding fluorescent stains such as chlortetracycline, Indo-1, Fura2, or their acetoxymethyl (AM) ester forms that allow for a free passive passage through the plasma membrane, have been used for decades for visualization and quantification of intracellular Ca^2+^ (Bush and Jones, 1987; Overvoorde and Grimes, 1994; Ramakrishna et al., 2011). Although informative, some of these dyes have limited cell penetration and preclude *in vivo* Ca^2+^ observation. Alternatively, FluoForte is an AM ester, Ca^2+^-binding fluorescent probe that solves some of the problems of previous probes and has proven useful to detect Ca^2+^ changes at specific time points. Using FluoForte to study microspore embryogenesis in the high response *B. napus* DH4079 line, it was shown that Ca^2+^ levels at the stages most sensitive to embryogenesis induction are higher than at earlier or later stages, and they increase even more just during the HS, and then decrease (Rivas-Sendra et al., 2017). Conversely, in microspores isolated from low-response materials like eggplant or the *B. napus* DH12075 line, Ca^2+^ levels are lower than in DH4079 microspores (Rivas-Sendra et al., 2019). Thus, there is a clear relationship between Ca^2+^ levels and embryogenic competence.

**Figure 1:**
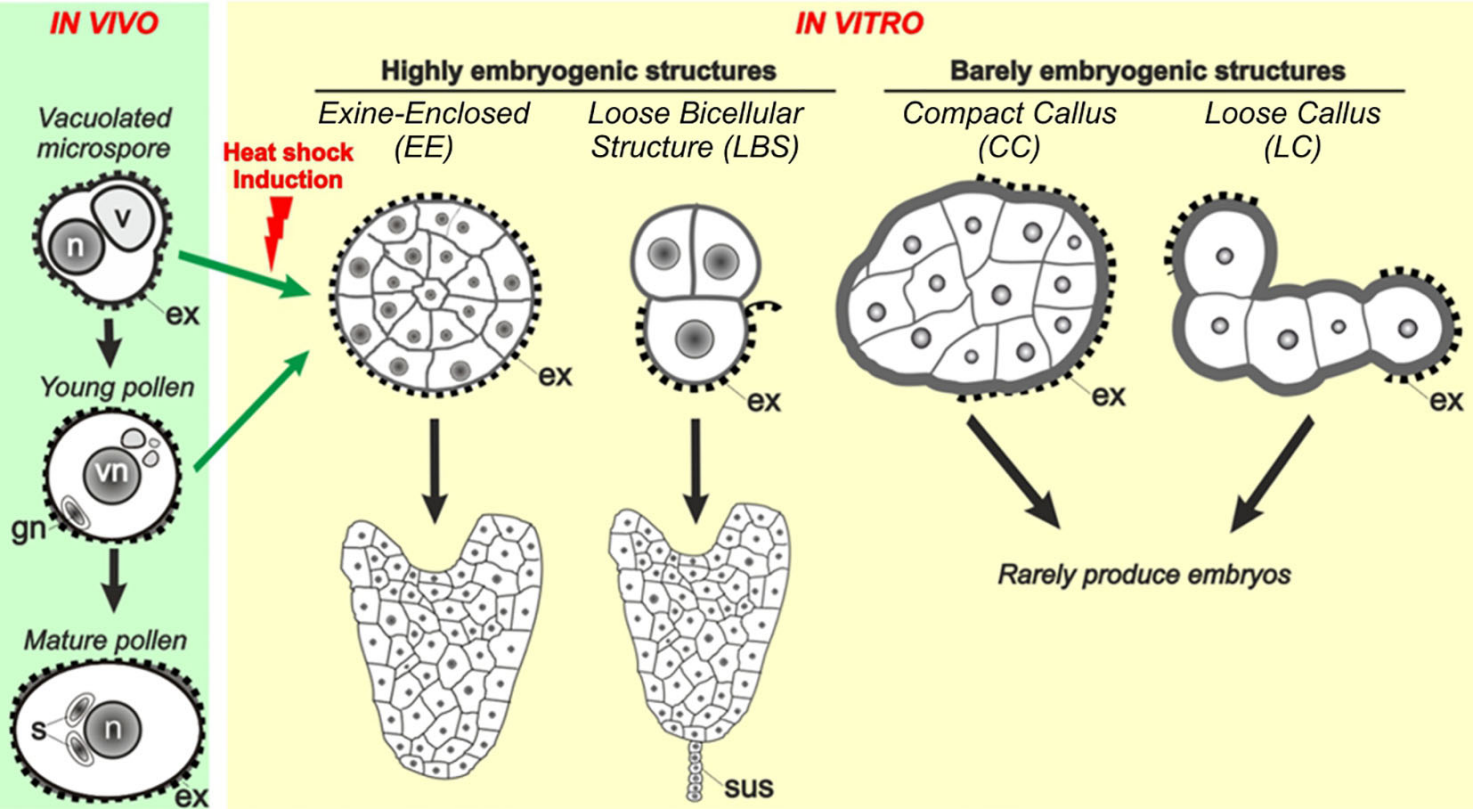
Different embryogenic structures in *B. napus* microspore cultures. Four different types of embryogenic structures are formed upon induction. They include exine-enclosed (EE) structures, loose bicellular structures (LBS), compact callus (CC) and loose callus (LC). EE and LBS are considered highly embryogenic structures as they have a high potential to become differentiated embryos, whereas CC and LC are barely embryogenic structures due to their very low potential to become embryos (Corral-Martinez et al., 2020). As opposed to embryos derived from EE structures, those derived from LBS develop a suspensor (sus), as zygotic embryos.

More recently, time-lapse imaging experiments (Corral-Martinez et al., 2020) revealed that few days after induction, *B. napus* microspores transform into four types of embryogenic structures (**Figure 1**): (1) exine-enclosed (EE) structures, which are abundant, globular and compact structures fully surrounded by exine; (2) loose bicelular structures (LBS), which are much less frequent embryogenic structures formed by two usually asymmetrically divided cells, sometimes with exine breaks, which soon differentiate into suspensor-bearing embryos (SUS); (3) compact callus (CC), abundant irregular cell masses with the exine broken and sometimes detached; and (4) loose callus (LC), callus masses characterized by their very irregular morphology, very low intercellular adhesion and extended areas devoid of exine. Irrespective of their frequency, each structure has different potential to become embryo, being EE and LBS/SUS considered highly embryogenic as many of them transform into viable embryos, whereas CC and LC are considered barely embryogenic because they never or very rarely, respectively, become embryos (Corral-Martinez et al., 2020). These four types of structures are also induced from DH4079 microspores, and specific cell wall features and responses to inhibition of histone deacetylases were found associated to their different embryogenic competences (Camacho-Fernández et al., 2021, 2024). Despite the clear relationship between Ca^2+^ and embryogenic competence, there are no clues about a possible involvement of Ca^2+^ in the occurrence of each different structure, in their different embryogenic potential, or in the modulation of their final embryo yield.

In this work, we studied Ca^2+^ dynamics using FluoForte staining during the different stages of microspore embryogenesis in *B. napus*, paying especial attention to the stages when the different embryogenic structures are formed, and how Ca^2+^ is distributed in their different cell domains. We also performed pharmacological studies to analyze the effect of modulating Ca^2+^ homeostasis at different stages of the process. We added to the culture medium different chemicals to increase intracellular and extracellular Ca^2+^ levels. To increase extracellular Ca^2+^ levels, Ca(NO_3_)_2_ was exogenously applied at concentrations higher than the typical concentration used in the standard *B. napus* culture medium. To increase intracellular Ca^2+^ levels, we applied ionophore A23187, a Ca^2+^-permeable membrane channel (Dedkova et al., 2000), inositol 1,4,5-trisphosphate (InsP_3_), and cyclopiazonic acid (CPA). InsP_3_ is known to promote Ca^2+^ release from intracellular stores (Im et al., 2010) to activate a series of developmental processes in plant and animal cells, including the endosperm central and egg cells (Han et al., 2002; Ge et al., 2007). CPA inhibits the ER Ca^2+^ pumps responsible for Ca^2+^ return from the cytoplasm to the ER after release (Ostry et al., 2018). We also used BAPTA-AM (the plasma membrane-permeable form of BAPTA) and EGTA (non-membrane permeable) to reduce the intracellular and extracellular Ca^2+^ levels, respectively. BAPTA and EGTA are two highly specific Ca^2+^ chelators (Tsien, 1980). Finally, we aimed to interfere with Ca^2+^ signaling with W-7 and CPZ, two calmodulin antagonists (Hidaka et al., 1981; Marshak et al., 1985), and with bepridil, a strong inhibitor of auxin-induced Ca^2+^ signaling (De Vriese et al., 2019). Altogether, these results show that Ca^2+^ levels are highly dynamic during microspore embryogenesis and intervene to modulate the final embryo yield.

## Materials and methods

### Plant material

DH4079, a high-response DH line (Corral-Martínez et al., 2021) selected from the *B. napus* Topas cultivar, was used for all the experiments. The low-response DH12075 line was also used to test the independent addition of Ca(NO_3_)_2_ and InsP_3_ to the culture medium. In both cases, plants were grown in 20 cm pots in a growth chamber at a 16/8 h photoperiod, 300 μE.m^−2^.s^−1^ light intensity, and 20°C during their vegetative growth period. Upon blooming, plants were transferred to chambers at 10°C with the same photoperiod and light intensity. Flower buds were collected at least one week after transference.

### Microspore culture

Microspore cultures were performed according to Corral-Martínez et al. (2021). Flower buds were collected from donor plants, measured and separated by size in three different ranges: 3.0-3.1, 3.2-3.3 and 3.4-3.5 mm. Microspores of the three ranges were processed and cultured in parallel, and only results from the best responding range were considered. Flower buds were transferred to tea sieves and surface-sterilized in the laminar flow hood by submerging them in 70% ethanol for 30 s and 10% bleach solution for 10 min. Then, floral buds were rinsed three times in sterile distilled water and transferred to three sterile 50 ml glass beakers. Filtered NLN-13, consisting of NLN salts and vitamins (Nitsch and Nitsch, 1967, Duchefa, Netherlands) supplemented with 130 g/L sucrose, pH 5.8, was used to isolate microspores. Buds were crushed with a sterile syringe piston to release microspores in NLN-13. The resulting microspore suspension was then filtered through a 40 µm nylon filter, and washed two times with 10 mL of NLN-13 medium. For this purpose, tubes containing microspore suspensions were centrifuged (100 g, 4 min) at 4°C in a refrigerated centrifuge. Finally, pelleted microspores were resuspended in 1 ml NLN-13 medium and the microspore density was estimated using a Improved Neubauer chamber as described (Camacho-Fernández et al., 2018). The final volume was adjusted by adding NLN-13 medium up to a density of 20.000 microspores/mL, and 500 µl of suspension were plated in each well of 24-well sterile plates. The induction treatment consisted of a 32°C heat shock for 3 days, after which plates were transferred to 25°C. Microspores were kept in darkness for the whole *in vitro* culture process. Embryo yield was measured by counting the total number of embryos in each well after one month in culture.

### Fluorescence and confocal microscopy

FluoForte (Enzo Life Sciences) staining was used to observe Ca^2+^ in microspores as described in Rivas-Sendra et al. (2017). Briefly, microspores were centrifuged (4 min, 200 g, room temperature), resuspended in PBS and centrifuged again. Precipitated microspores were resuspended in equal volumes of PBS and 0.2 g/L FluoForte solution. Samples were incubated 30 min in darkness, washed with PBS and centrifuged (2 min, 200 g). Microspores were then mounted in microscope slides with Mowiol anti-fading mounting solution made with 17% Mowiol 4–88 (Sigma-Aldrich) and 33% glycerol (v/v) in PBS. Samples were observed in a Zeiss 780 Axio Observer confocal microscope using an excitation wavelength of 488 nm and recording emission at 516 nm, and in a Nikon E1000 fluorescence microscope.

### Quantification of size and fluorescence intensity

Quantification of area and FluoForte-specific fluorescence intensity was performed using the FIJI software (Schindelin et al., 2012). For each studied structure, paired bright field and fluorescence images were taken with a Nikon Eclipse E1000 fluorescence microscope. The perimeter, excluding the exine coat, was delineated in the bright field image, generating a Region of Interest (ROI) that was transferred to the fluorescence image, where area and signal intensity were estimated using the *Area* and *Mean gray value* tools. Day-3 microspores were discriminated by size using a threshold of 720 µm^2^, which is the area of the largest microspore measured at day 0, prior to induction. Thus, day-3 microspores with areas lower than 720 µm^2^ were considered as not growing, since after 3 days they did not exceed the size of the largest day-0 microspore. Conversely, microspores with areas higher than 720 µm^2^ were considered as growing. The Kruskal-Wallis non-parametric test (p ≤ 0.05) was used to determine differences between medians. Paired comparisons among samples were performed using the Bonferroni procedure (p<0.05). All images were taken under the same experimental conditions and all fluorescence intensity measurements were made under identical software conditions.

### Chemical treatments

Ca(NO_3_)_2_ was used as an additional source of exogenous Ca^2+^. Ionophore A23187 was used as a Ca^2+^ channel to alter Ca^2+^ gradients. To release intracellular Ca^2+^, InsP_3_ was used. 1,2-bis(2-aminophenoxy)ethane-N,N,N’,N’-tetra acetic acid tetrakis acetoxymethyl ester (BAPTA-AM) and ethylene glycol-bis(β-aminoethyl ether)-N,N,N′,N′-tetra acetic acid (EGTA) were used as selective chelators of intracellular and extracellular Ca^2+^, respectively. N-(6-Aminohexyl)-5-chloro-1-naphthalenesulfonamide hydrochloride (W-7) and chlorpromazine hydrochloride (CPZ) were used as calmodulin inhibitors. Cyclopiazonic acid (CPA) was used as an inhibitor of ER Ca^2+^ pumps. Bepridil was used as a blocker of Ca^2+^ channels involved in auxin-mediated Ca^2+^ signaling. All chemicals were purchased from Sigma-Aldrich except for BAPTA-AM (Abcam) and bepridil (Enzo Life Sciences). Stocks were prepared according to product specifications, dissolved in water (Ca(NO_3_)_2_, CPZ, EGTA, and InsP_3_), ethanol (bepridil) or DMSO 2.6% (ionophore A23187), 1% (BAPTA-AM), 10% (W-7) or 100% (CPA). They were added to microspore cultures in appropriate volumes of the stock solutions for the final concentrations described in Results. For chemicals where DMSO or ethanol was used to prepare stocks, controls were prepared by adding the same amount of DMSO or ethanol added together with the corresponding chemical. All compounds were added at the time of culture initiation (day 0) and removed after 3 days, 7 days or one month (continuous exposure), which is when cultures in all cases were finished and embryos counted. At least three biological replicates were performed for each experiment.

Embryo yield typically shows variability among cultures. In order to facilitate comparisons among treatments, the results of chemical treatments were expressed normalizing the embryo yield of DH4079 controls to the reference value of 100, and then calculating the corresponding values of the treatments. For the experiments with the low-response DH12075 line, embryo yield values were normalized to 10. The absolute values of these experiments are shown in **Supplementary Table S1**. One-way ANOVA test (p ≤ 0.05) was performed to determine statistical differences among culture conditions. Then, significance groups were established through the Least Significance Difference (LSD) method. To estimate the percentages of the different embryogenic structures (EE, LBS/SUS, CC and LC), experiments were repeated using the optimal concentration of each chemical. For chemicals with positive effects in microspore embryogenesis, the optimal concentration is defined as the concentration producing the highest embryo yield, whereas for chemicals with negative effects, it is defined as the maximum concentration with a non-null effect. For all experiments, the percentages of the four types of structures were calculated by counting a minimum of 200 structures at day 6 of culture.

## Results

### Occurrence of different types of embryogenic structures in *Brassica napus* microspore culture

**Figure 2:**
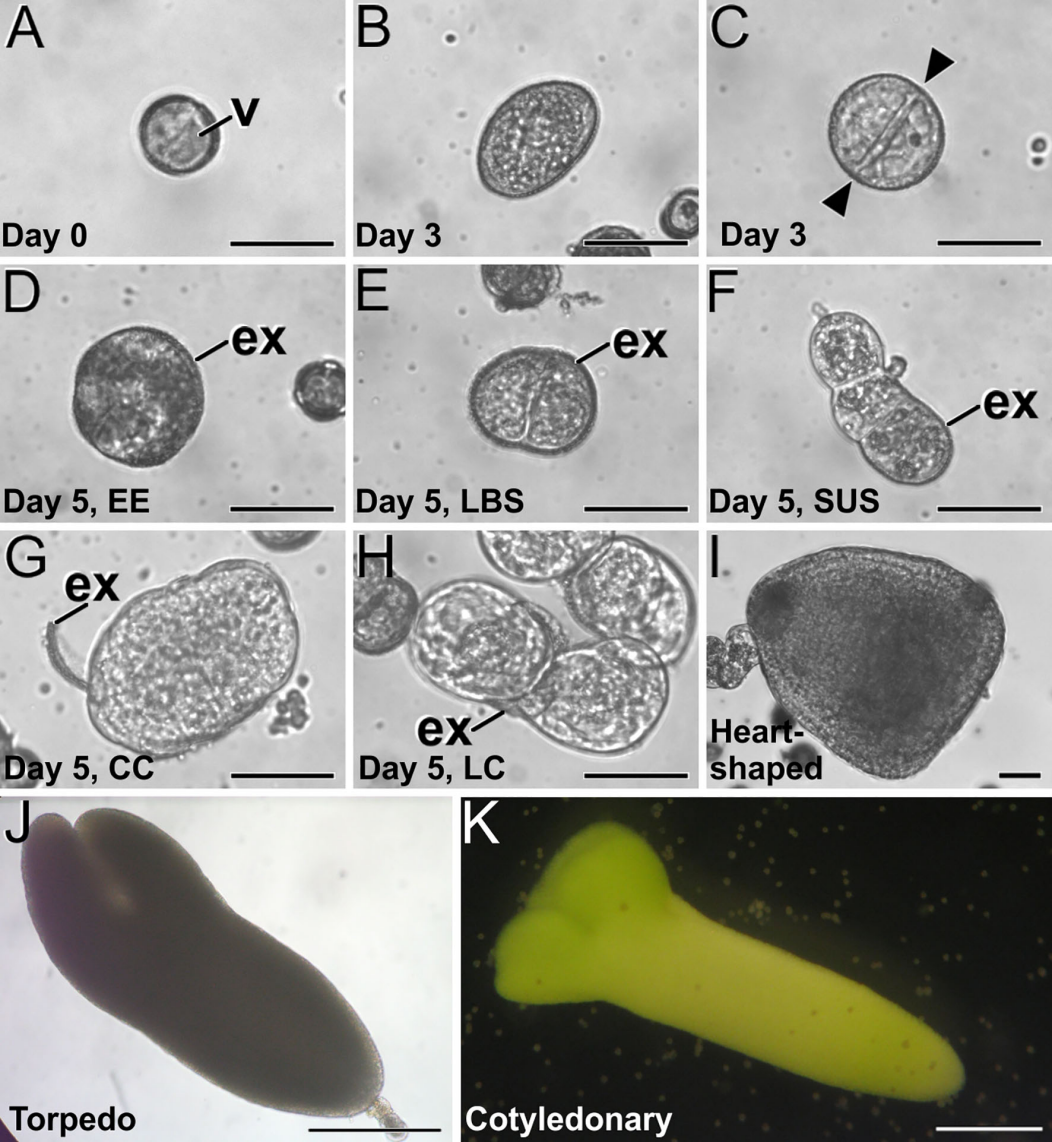
*B. napus* microspore culture. **(A)** Freshly isolated vacuolated microspore before induction. **(B, C)** Three-day-old cultures, just after induction, showing a non-induced, pollen-like structure **(B)** and an induced, embryogenic structure **(C)** where an equatorial division is clearly observed (arrowheads). **(D–H)** Five-day-old cultures where four distinct embryogenic structures can be distinguished, including an EE structure **(D)**, an LBS still mostly covered by exine **(E)** and transformed into an early suspensor (SUS) embryo **(F)**, a CC **(G)** and a LC structure **(H)**. **(I)** Two-week-old suspensorless heart-shaped embryo. **(J)** Three-week-old suspensor-bearing torpedo embryo. **(K)** One-month-old cotyledonary embryo. ex, exine; gn, generative nucleus; n, nucleus; s, sperm cells; sus, suspensor; v, vacuole; vn, vegetative nucleus. Bars: **(A–I)**: 50 µm; **(J, K)** 200 µm.

Microspore culture starts with the isolation of vacuolated microspores and young pollen grains and their inoculation in the culture medium (**Figure 2A**) for application of the 3-day-long 32°C HS treatment. After this time, some microspores/pollen are not sensitive to the induction treatment and developed into pollen-like structures (**Figure 2B**) whereas others become induced, as evidenced by their enlargement and the occurrence of the first equatorial divisions (**Figure 2C**). At this culture time, no clear morphological differences among embryogenic structures could be detected. Five days after culture initiation, however, the differentiation of four distinct embryogenic structures was evident. As previously described in Camacho-Fernández et al. (2021), we observed compact EE structures (**Figure 2D**), LBS (**Figure 2E**) which developed into SUS embryos (**Figure 2F**), disorganized CC structures with exine detached from the structure (**Figure 2G**) and LC structures, with loosely connected cells almost devoid of exine (**Figure 2H**). From day 8 on, both globular embryos and callus structures kept growing and heart-shaped (**Figure 2I**), torpedo (**Figure 2J**) and cotyledonary embryos (**Figure 2K**) were observed. This experimental system was used in all the studies presented next.

### Ca^2+^ distribution in the different microspore-derived embryogenic structures

**Figure 3:**
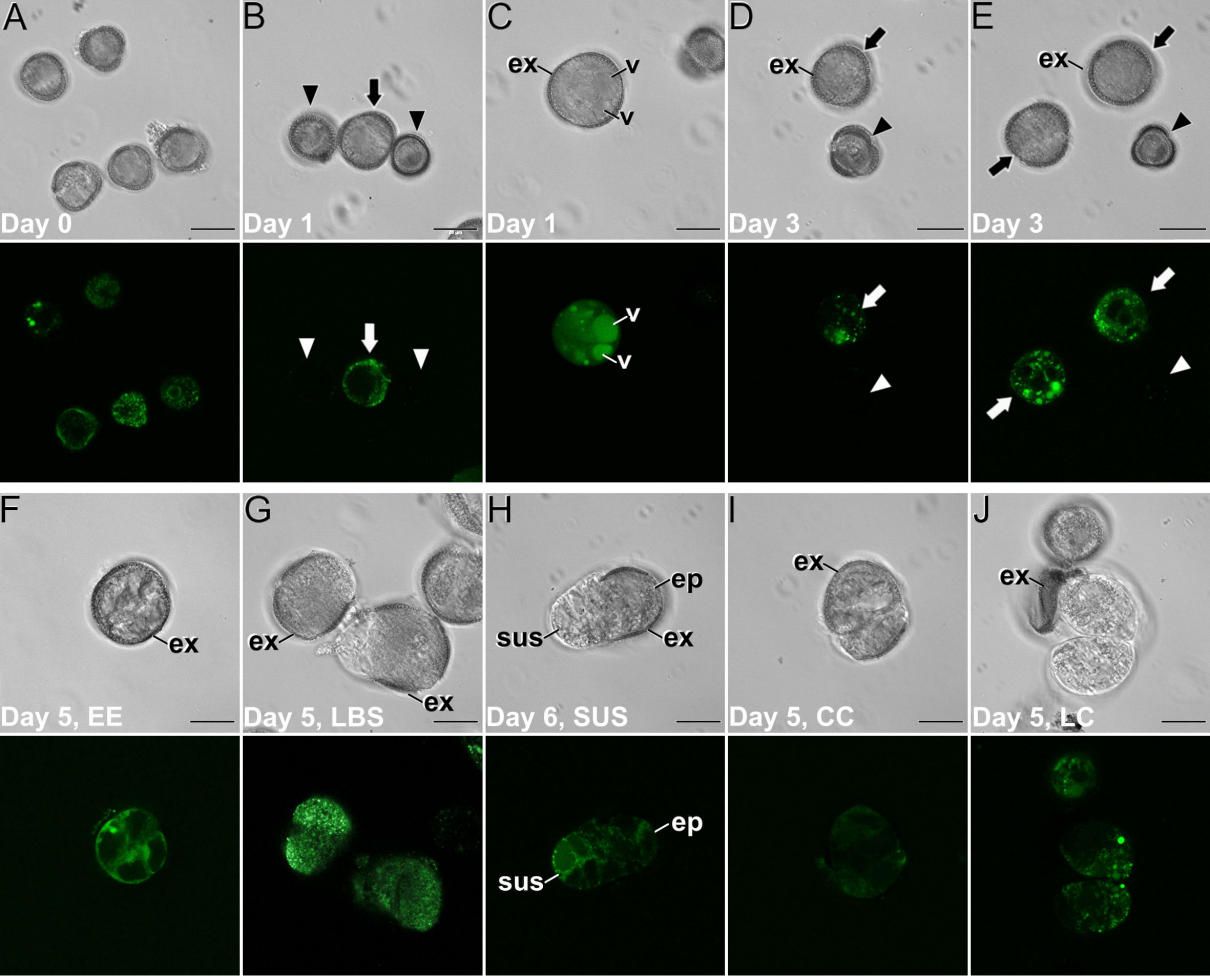
Ca^2+^ detection with FluoForte in *(B) napus* microspore cultures. Paired images of the same microscopic field imaged by phase contrast optics (top image) and fluorescence (bottom image). **(A)** Freshly isolated, vacuolated microspores. **(B)** One-day-old culture showing an enlarged, growing microspore (arrow) with an intense FluoForte signal, and arrested microspores with no visible signal (arrowheads). **(C)** One-day-old enlarged, microspore with intense FluoForte signal in all the cell, and principally in the vacuoles (v). **(D, E)** Three-day-old induced embryogenic structures (arrows) with different FluoForte signal intensities, together with arrested microspores with no FluoForte staining (arrowheads). **(F–J)** Images of fiveday-old cultures showing an EE structure **(F)**, two LBS **(G)** still mostly covered by exine (ex), a suspensor-bearing embryo (H) where the suspensor (sus) and embryo proper (ep) domains with different fluorescence intensities can be distinguished, a CC **(I)** and a LC structure **(J)**. Bars: 20 μm.

**Figure 4:**
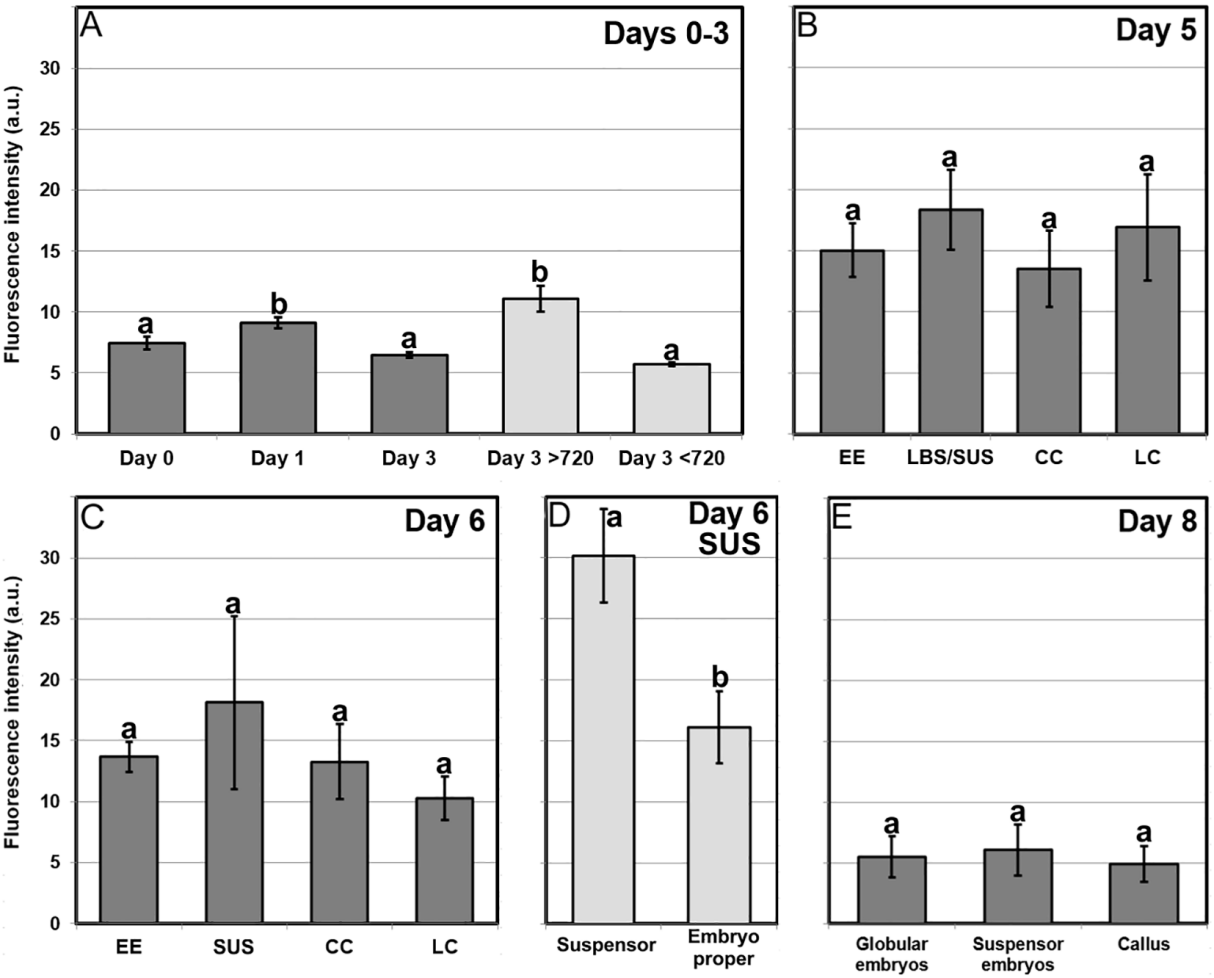
Quantification of FluoForte-signal in *B. napus* microspore cultures. Fluorescent signal intensity is expressed in arbitrary units (a.u.) ± standard error (error bars). **(A)** Quantification of FluoForte signal in microspores of day 1, day 2 and day 3 cultures. Light grey bars represent a segregation of total day 3 structures in two categories: structures smaller and larger than 720 μm^2^ (see text for further details). **(B, C)** Quantification of FluoForte signal in the different embryogenic structures (EE, LBS/SUS, CC and LC) identified in five **(B)** and six-day-old cultures **(C)**. **(D)** Quantification of FluoForte signal in each of the suspensor and embryo proper domains of the suspensor-bearing structures (SUS) observed at day six. **(E)** Quantification of FluoForte signal in the different suspensorless embryos, suspensor-bearing embryos and calli observed in eight-day-old cultures. All fluorescence intensity measurements were made under identical experimental conditions and are represented using the same scale. For each chart, different letters indicate significant differences according to the Kruskal-Wallis test (p ≤ 0.05).

We studied Ca^2+^ levels and distribution by FluoForte staining and observation at the confocal microscope through the different stages of microspore culture, paying special attention to the different embryogenic structures developed from induced microspores (**Figure 3**; **Supplementary Figure S1**). We also performed a quantitative study of fluorescence intensity (**Figure 4**) and size (**Supplementary Figure S2**) of the different structures stained with FluoForte and observed at each stage with the fluorescence microscope. In freshly isolated microspores, prior to the application of the heat shock (**Figure 3A**), FluoForte staining was high in vacuolated microspores and young pollen grains, as previously described (Rivas-Sendra et al., 2017), whereas in other, younger stages, the FluoForte signal was negligible. One day after initiation of induction, FluoForte staining was intense in the cytoplasm and nucleus (**Figure 3B**, arrow) of enlarged, growing microspores whereas in other microspores with no visible signs of growth, the FluoForte signal was much lower or even absent (**Figure 3B**, arrowheads). In the most enlarged microspores, the FluoForte signal began to accumulate in the vacuoles (**Figure 3C**). Three days after induction, most microspores showed very low or no signal (**Figures 3D, E**, arrowheads), but some of them still presented fluorescence with variable intensities, ranging from similar to day 0 (**Figure 3D**, arrow) to much higher (**Figure 3E**, arrow). Due to this variability, we discriminated day-3 microspores in two size-based categories as described in Materials and methods, considering those with an area lower than 720 µm^2^ as not growing, and those larger than 720 µm^2^ as growing, and therefore possibly embryogenic but not yet differentiated into any identifiable embryogenic structure. The fluorescence intensity of >720 µm^2^ microspores doubled that of <720 µm^2^ microspores (**Figure 4A**), thereby confirming the tight relationship between Ca^2+^ accumulation and embryogenic cell growth at early stages. At days 5 and 6 of culture (**Figures 3F–J**), when the four types of embryogenic structures were easily identifiable, all of them showed an increased size (**Supplementary Figure S2B**) and FluoForte signal intensity, being 2-3 times higher than in isolated microspores. In 6-day-old cultures, the different embryogenic structures were slightly larger, principally callus structures (CC and LC; **Supplementary Figure S2C**). However, the faster growth of these structures was not correlated with their pattern of FluoForte staining, which was almost identical to day 5. There were not significant differences in intensity among the different types of structures (**Figures 4B, C**). In slightly more advanced LBS structures, when they transform into suspensor-bearing (SUS) embryos, suspensor cells presented fluorescence levels higher than cells of the embryo proper (**Figure 3H**), as reflected in the comparatively higher standard error for LBS/SUS structures (**Figure 4C**). This led us to calculate separately the fluorescence intensities of each cell, confirming that suspensor cells have approximately twice the intensity of embryo proper cells (**Figure 4D**), which suggests a link between Ca^2+^ accumulation and suspensor cell identity. From day 8 on, both the embryos produced from highly embryogenic structures (**Supplementary Figures S1A, B**) and the calli produced from barely embryogenic structures (**Supplementary Figure S1C**) presented in general low levels of FluoForte staining (**Figure 4E**), much lower than their precursors (**Figure 4C**) and comparable to freshly isolated microspores (**Figure 4A**). In conclusion, both qualitative and quantitative analysis of Ca^2+^ dynamics with FluoForte revealed that induction of embryogenesis is associated with a progressive increase in Ca^2+^ levels just after HS application until days 5-6. This is the period when embryogenic microspores grow and differentiate into the four types of embryogenic structures. Then, there is a decrease of Ca^2+^ levels, reaching the initial, day 0 levels in 8-day calli and embryos.

### Effects of increasing Ca^2+^ availability

**Figure 5:**
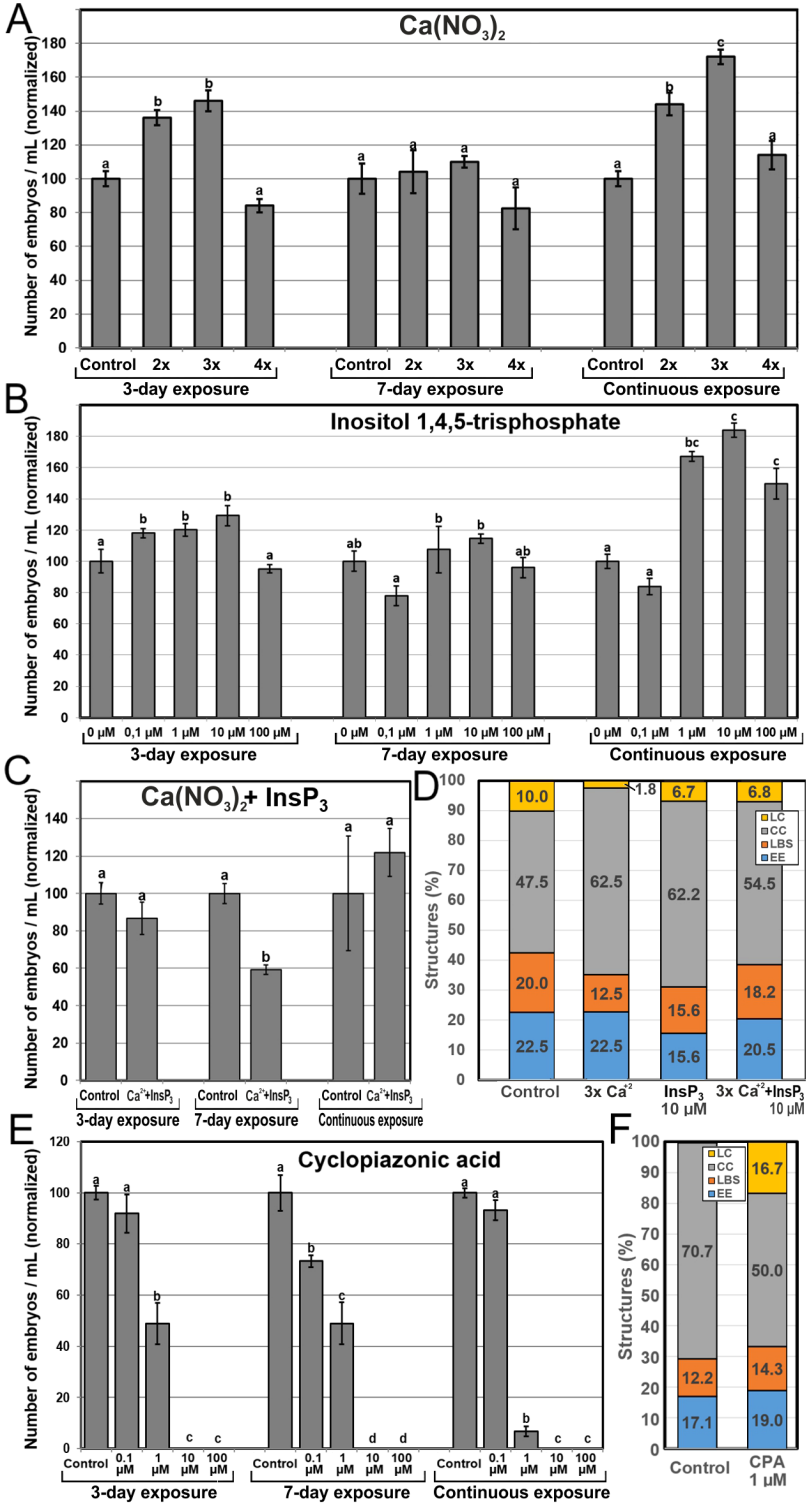
Effects of increasing Ca^2+^ availability. Ca^2+^ availability was increased with the independent addition to the culture medium of different concentrations of Ca(NO_3_)_2_ **(A)**, InsP_3_ **(B)**, and a combination of 3x Ca(NO_3_)_2_ and 10 µM InsP_3_ **(C)**. For Ca(NO_3_)_2_, 2x, 3x and 4x represent two, three or four times the standard Ca(NO_3_)_2_ concentration used in control cultures (500 mg/L). **(D)** Changes in the percentages of embryogenic structures produced with the independent addition during 6 days of 3x Ca(NO_3_)_2_ **(A)**, 10 µM InsP_3_ **(B)**, and a combination of 3x Ca(NO_3_)_2_ and 10 µM InsP_3_. **(E, F)** Changes in the number of embryos **(E)** and the percentage of embryogenic structures **(F)** produced with the addition of cyclopiazonic acid (CPA) in the same conditions described above. For **(A–C, E)** the different chemicals and concentrations were applied during the first three days of culture, during seven days, and continuously, and effects are expressed as number of embryos produced per mL of culture medium, normalizing control values to 100. Different letters indicate significant differences according to the LSD test (p ≤ 0.05). For **(D, E)**, chemicals were applied at their optimal concentration and the different embryogenic structures produced were counted at day 6 and expressed as percentages.

Once established the correlation between Ca^2+^ levels and microspore embryogenesis, we performed a pharmacological study to modulate the intracellular Ca^2+^ levels with different chemicals known to interfere with Ca^2+^ levels and signaling. Chemicals were applied at different concentrations and exposure times, and their effects were evaluated by counting the number of embryos produced by each treatment after 30 days of culture. First, we aimed to increase the levels of available Ca^2+^ by adding to the cultures: (1) increased Ca(NO_3_)_2_ concentrations, (2) InsP_3_, to release Ca^2+^ from intracellular stores, and (3) ionophore A23187, a plasma membrane-intercalating Ca^2+^ channel. First, we added extra Ca(NO_3_)_2_ to the culture medium at concentrations corresponding to 2, 3 and 4-fold the regular Ca(NO_3_)_2_ concentration (500 mg/L) present in the NLN medium used in control cultures (**Figure 5A**). When applied during the first 3 days of culture, 2x and 3x Ca^2+^ concentrations significantly increased embryo production up to 40%. No differences were found with any Ca^2+^ concentration at 7-day application, but for continuous exposure, there were significant and dose-dependent increases in embryo yield of up to 70% that of control cultures (**Figure 5A**). Addition of InsP_3_ (**Figure 5B**) resulted in a similar pattern in terms of embryo yield. The number of embryos was 20-30% higher than in controls using 0.1, 1 and 10 µM InsP_3_ when applied during the first 3 days of culture, and up to 80% higher when applied continuously at 1 and 10 µM (**Figure 5B**). No significant differences were observed when InsP_3_ was applied for 7 days. With the addition of ionophore A23187, embryo yield was drastically reduced or null with all the exposure times and concentrations used (**Supplementary Figure S3**), suggesting either the use of an excessively high concentration range or any sort of technical problem with the batch used. Anyway, considering the positive results obtained with Ca(NO_3_)_2_ and InsP_3_, we focused on them and discarded ionophore A23187 for further experiments. Ca(NO_3_)_2_ and InsP_3_ showed that embryo production is favored when either extracellular or intracellular Ca^2+^ levels are increased during the first 3 days or continuously, but not during days 1-7. Increased Ca^2+^ levels during days 4-7 seem to prevent embryogenic differentiation, as they compensate the positive results of increasing Ca^2+^ during days 1-3 for a net result of no significant differences.

Due to the positive effects in embryo yield of specific Ca(NO_3_)_2_ and InsP_3_ combinations of concentration and time, we explored possible synergistic effects with the combined application of Ca(NO_3_)_2_ and InsP_3_ at 3x and 10 µM, their respective optimal concentrations (**Figure 5C**). No positive results were observed. Instead, application during 3 days of culture and continuous application resulted in no significant differences with respect to control conditions. Interestingly, when applied for 7 days, embryo yield decreased versus control conditions. Thus, the positive independent effects of adding Ca(NO_3_)_2_ and InsP_3_ during the first three days and continuously disappeared when added together, being even negative when applied during 7 days.

In order to understand the relationship between Ca^2+^ and increased embryo yield, we performed new microspore cultures adding 3x Ca(NO_3_)_2_, 10 µM InsP_3_, and a combination of both, and counted the percentages of each type of embryogenic structure produced by each treatment at day 6 (**Figure 5D**). The addition of 3x Ca(NO_3_)_2_ led to a reduction of 18% in the percentage of highly embryogenic structures (EE+LBS/SUS), due principally to a 38% reduction in the percentage of LBS/SUS, whereas the addition of 10 µM InsP_3_ caused a reduction of 27% in the percentage of highly embryogenic structures (EE+LBS/SUS), due principally to a 31% reduction in the percentage of EE (**Figure 5D**). However, the combined application of InsP_3_ and Ca(NO_3_)_2_, which had negative effects in embryo yield, produced very limited reductions of the percentages of highly embryogenic structures. Thus, the increases in embryo yield observed with the independent use of Ca(NO_3_)_2_ and InsP_3_ are not related to an increase in the proportion of highly embryogenic structures present in early culture stages.

We next tested the positive results of independent InsP_3_ and Ca(NO_3_)_2_ addition in the low-response *B. napus* DH12075 line. Addition of InsP_3_ at any concentration or exposure time did not produce any significant change compared to controls. Addition of Ca(NO_3_)_2_ (**Supplementary Figure S4**) showed no significant differences versus control when applied during the first three days. However, the addition of 2x and 3x the standard Ca(NO_3_)_2_ concentration during the first 7 days of culture produced five times more embryos than controls, although their size was smaller that the DH4079 counterparts. When applied continuously, the results were negative at all concentrations. Thus, the exogenous addition of Ca^2+^ for 7 days can be used to increase the embryogenic response also in the low response genotype.

### Effects of blocking ER Ca^2+^ pumps

We used CPA to block ER Ca^2+^ pumps, precluding the return of intracellular Ca^2+^ levels to those previous to Ca^2+^ release. In general, embryo yield was negatively affected by CPA (**Figure 5E**) irrespective of the exposure time, and in a dose-dependent manner, being not significantly affected at low concentrations, largely reduced at mid-range concentrations, and null at the highest concentrations. The percentages of highly embryogenic structures were similar to those of controls (**Figure 5F**), with little individual differences between EE and LBS/SUS. The overall percentages of barely embryogenic structures were also similar to control, but a remarkable transition from CC to LC structures was evidenced. Thus, the blockage of Ca^2+^ translocation back to the ER reduced embryo production, in a dose-dependent manner, by reducing the number of induced, dividing microspores but not the percentage of highly embryogenic structures. These results demonstrate that a proper recovery after Ca^2+^ release is essential for proper embryo induction and development.

### Effects of reducing Ca^2+^ availability

**Figure 6:**
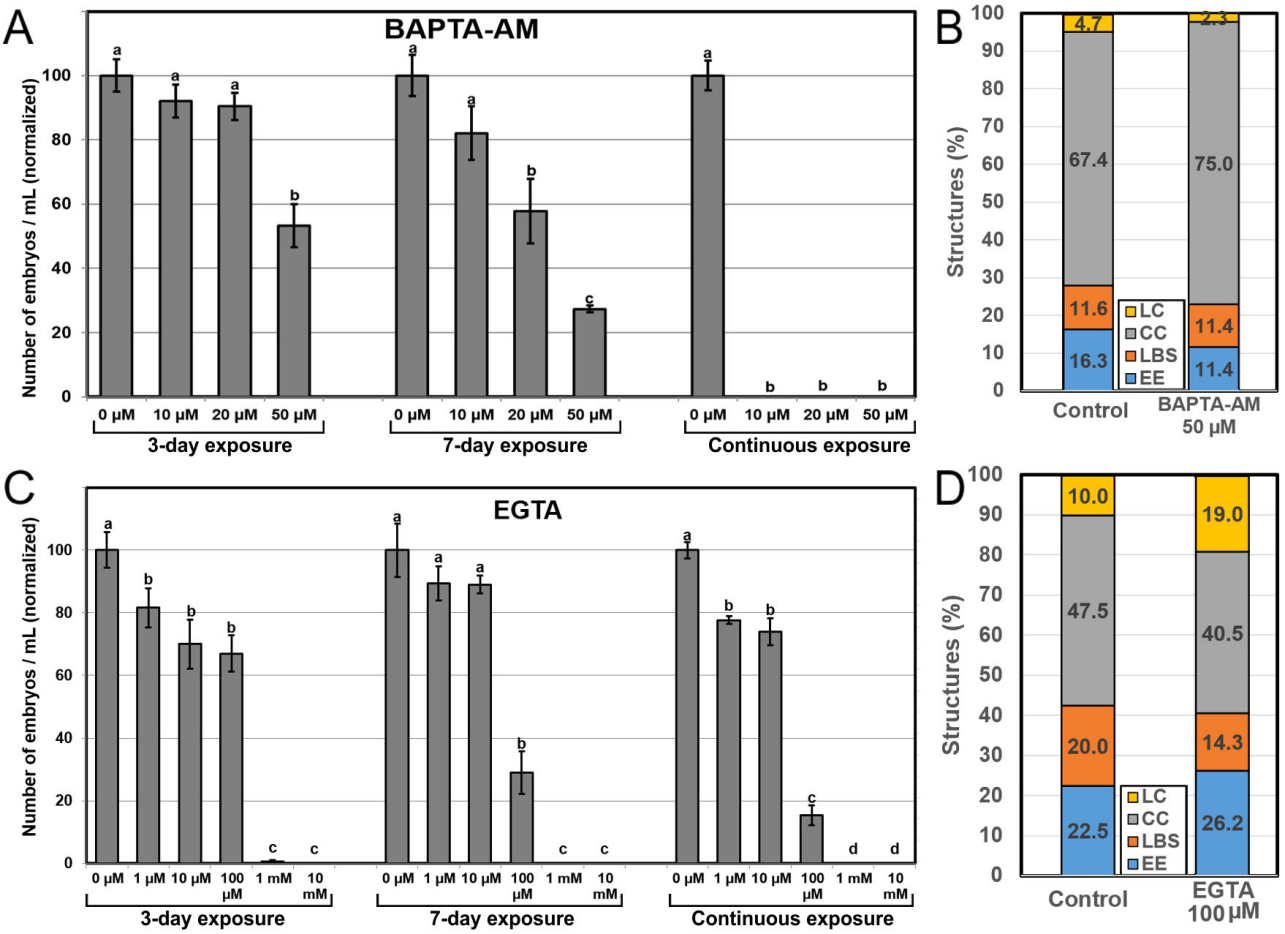
Effects of reducing Ca^2+^ availability. Ca^2+^ availability was reduced with the independent addition to the culture medium of different concentrations of BAPTA-AM **(A, B)** and EGTA **(C, D)**. For **(A, C)**, the different chemicals and concentrations were applied during the first three days of culture, during seven days, and continuously, and effects are expressed as number of embryos produced per mL of culture medium, normalizing control values to 100. Different letters indicate significant differences according to the LSD test (p ≤ 0.05). For **(B, D)**, chemicals were applied at their optimal concentration and the different embryogenic structures produced were counted at day 6 and expressed as percentages.

Next, we assessed the effects of reducing intracellular and extracellular Ca^2+^ availability with the use of two Ca^2+^ chelators, BAPTA-AM and EGTA, respectively. Application of BAPTA-AM (**Figure 6A**) during the first three days of culture had only a negative effect in embryo production at 50 µM, the highest concentration (~50% reduction vs control). Application for seven days evidenced negative effects even with lower concentrations, and the effects with higher concentrations were more severe (~67% reduction *vs* control for 50 µM). Continuous application completely inhibited embryo production at all concentrations. These time and dose-dependent negative effects of BAPTA-AM were not anticipated by a dramatic alteration of the percentages of the different embryogenic structures formed at day 6 of culture, which were remarkably similar to those found in controls (**Figure 6B**). Exposure to EGTA to reduce extracellular Ca^2+^ levels had similar dose-dependent effects, being slightly negative at low concentrations, severely negative at mid-range concentrations, and completely inhibiting embryo production at the highest concentration tested (**Figure 6C**). However, only the mid range 100 µM concentration showed a time-dependent effect, as the reduction of embryo yield *vs* control was ~33% for 3-day, ~70% for 7-day and ~85% for continuous exposure. As with BAPTA-AM, EGTA did not alter the percentages of the different embryogenic structures at day 6, which were similar to those of controls except for LC structures, which nearly doubled (**Figure 6D**). This, however, did not represent a relevant change in the percentage of barely embryogenic structures. Together, these results confirm that chelation of intracellular and extracellular Ca^2+^ has similar time and dose-dependent negative effects in embryo yield. Although the optimal concentration for BAPTA-AM was higher than for EGTA (50 µM vs 100 µM), BAPTA-AM produced more severe effects than EGTA. However, as for the experiments to increase Ca^2+^ availability, changes in Ca^2+^ levels had no effect in the proportion of highly embryogenic structures formed.

### Effects of inhibiting Ca^2+^ signaling

**Figure 7:**
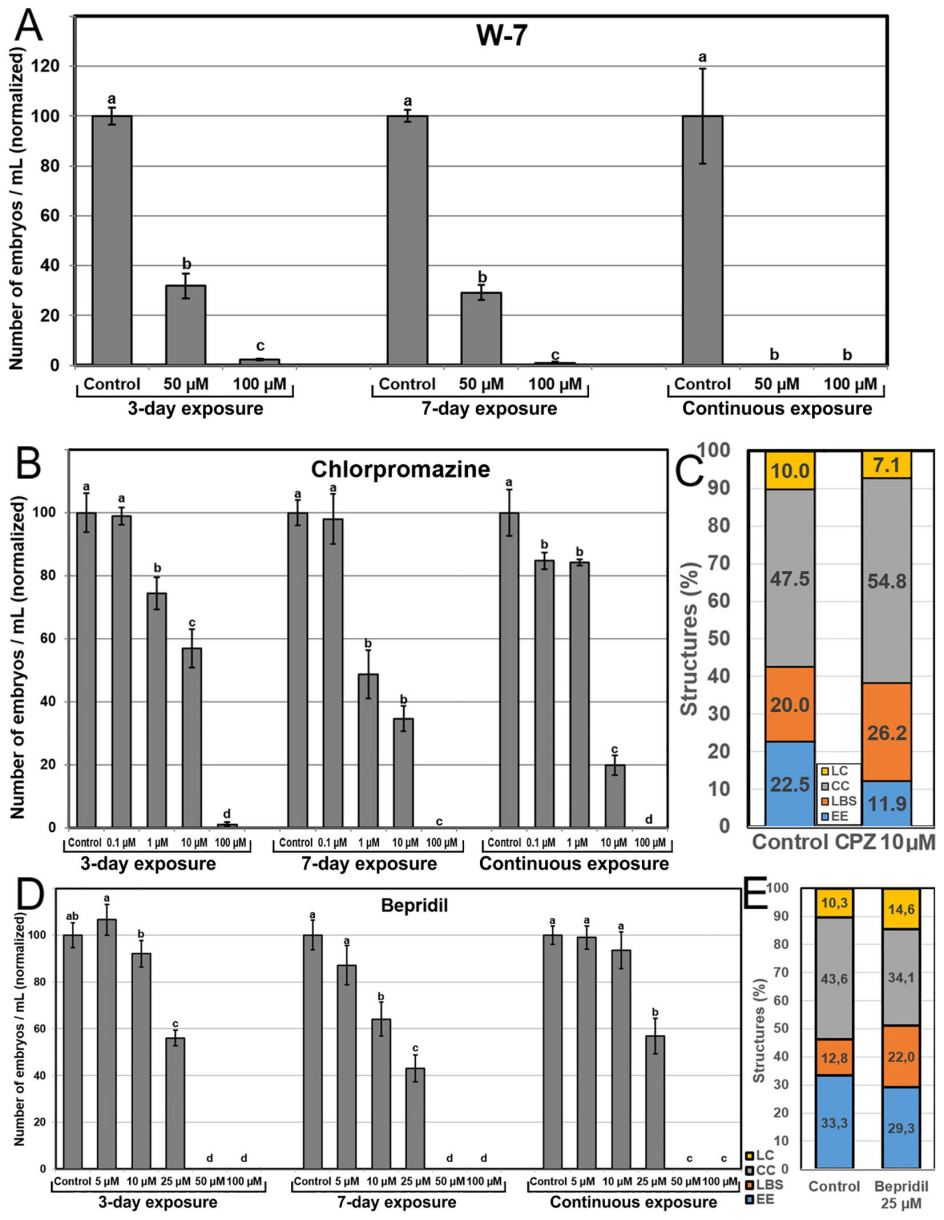
Effects of inhibiting CaM. CaM was inhibited with the independent addition to the culture medium of different concentrations of W-7 **(A)**, CPZ **(B, C)**, and bepridil **(D, E)**. For A, B and D, the different chemicals and concentrations were applied during the first three days of culture, during seven days, and continuously, and effects are expressed as number of embryos produced per mL of culture medium, normalizing control values to 100. Different letters indicate significant differences according to the LSD test (p≤0.05). For **(C)** and **(E)**, chemicals were applied at their optimal concentration and the different embryogenic structures produced were counted at day 6 and expressed as percentages.

Our next goal was to evaluate the effect of chemical inhibition of Ca^2+^ signaling. We used W-7, a CaM antagonist, CPZ, a CaM inhibitor, and bepridil, which blocks Ca^2+^ channels involved in auxin-mediated Ca^2+^ signaling. Application of W-7 severely affected embryo yield at 50 µM and was almost completely inhibited at 100 µM (**Figure 7A**). The effects were similar for 3 and 7-day applications, and more severe for continuous application, which led to a complete inhibition of embryo production at any concentration. CPZ (**Figure 7B**) had in general a clear dose-dependent negative effect. Low concentrations had little or no effect, intermediate concentrations drastically reduced embryo yield, and high concentrations produced almost no embryos. Although the percentage of EE structures formed at day 6 with 10 µM CPZ was reduced to ~50%, the overall percentage of highly embryogenic structures was not far from control (38.1% vs 42.5%), due to the increased percentage of LBS forms (**Figure 7C**).

Addition of bepridil produced a dose-dependent profile, with no effect at low doses, ~45-55% reduction of embryo yield at mid-range concentrations (25 µM), and complete inhibition of embryo production at higher concentrations (**Figure 7D**). The similarity of the 3-day, 7-day and continuous exposure profiles suggested that the main effect of bepridil in the inhibition of auxin-mediated Ca^2+^ signaling is produced during the first three days, just when microspores are being induced to embryogenesis. The percentages of the different embryogenic structures formed at day 6 with the optimal bepridil concentration (25 µM) showed an increase of the percentages of LC and, principally, of LBS structures, although the percentage of highly embryogenic structures (EE+LBS, 49.3%) was not different enough from control (46.1%) to explain the reduction in embryo yield (**Figure 7E**).

## Discussion

We showed that in the DH4079 line of *B. napus*, it is possible to induce the formation of embryogenic microspores from which, upon cessation of the HS, different types of embryogenic structures with different embryogenic potential (the highly embryogenic EE and LBS/SUS and the barely embryogenic CC and LC), are formed after 5 days of culture. This contrasts with previous reports showing the occurrence of the same embryogenic structures after just 3 culture days in both the high response DH4079 and the low response DH12075 lines (Li et al., 2014; Corral-Martinez et al., 2020; Camacho-Fernández et al., 2021, 2024). This difference relies on the fact that in these reports, a one-day-long HS was sufficient to induce a sufficient number of embryos. Although we have routinely been using the 1 day-32°C conditions (Corral-Martínez et al., 2013, 2019; Parra-Vega et al., 2015a, b; Rivas-Sendra et al., 2017, 2019; Camacho-Fernández et al., 2021, 2024), in some cases, 1 day is not enough to induce a high response, and the HS is extended for up to 3 days at 32°C to obtain similar results (Jouannic et al., 2001; Satpute et al., 2005; Rivas-Sendra et al., 2017). Even in some cases, 14 days at 30°C have successfully been used (Abdollahi et al., 2012). Temperature is key to determine the developmental fate of *B. napus* cultured microspores (Custers et al., 1994). Indeed, there is a clear correlation between HS duration and growth speed of the embryos produced (Corral-Martínez et al., 2021). In this work, the 3-day-long HS delayed the growth of the different embryogenic structures compared to previous reports. However, their anatomy, developmental features and FluoForte staining patterns were not affected, being equivalent to those of previous works, as described next.

### Cytoplasmic Ca^2+^ increase improves embryo yield by increasing the number of embryogenic microspores

We used higher concentrations of Ca(NO_3_)_2_ and InsP_3_ to increase the available extracellular and intracellular Ca^2+^ levels, respectively. Addition of Ca(NO_3_)_2_ to the culture medium increases the intracellular-extracellular Ca^2+^ gradient, thereby promoting Ca^2+^ influx and elevating cytosolic Ca^2+^ levels (Lecourieux et al., 2002; Takeda et al., 2003). In other *in vitro* embryogenesis systems (isolated carrot somatic cells), a 4 mM increase of Ca^2+^ concentration in the culture medium for 3 days was sufficient to enhance somatic embryo production from 18% to 58% (Calabuig-Serna et al., 2023a). InsP_3_ is a signal transduction intermediate that triggers the release of Ca^2+^ from intracellular stores, principally (but not only) the vacuole (Im et al., 2010), thereby activating the corresponding Ca^2+^-mediated signaling cascades (Berridge, 1993). Despite that plant homologues of animal InsP_3_ receptors have not yet been identified in higher plants (Edel et al., 2017), there is strong evidence for InsP_3_-induced Ca^2+^ release in many different plant processes (Krinke et al., 2006), including plant embryogenesis. In particular, InsP_3_ stimulates Ca^2+^ release similar to sperm cell-mediated activation of the endosperm central cell (Han et al., 2002) and the plant and animal egg cells (Ge et al., 2007). The similarities of these processes with the activation of the microspore to become embryogenic led us to assess the use of InsP_3_ in microspore embryogenesis. With both Ca(NO_3_)_2_ and InsP_3_, we obtained very similar results in DH4079: three-day applications significantly increased embryo yield (**Figures 5A, B**), which confirms that increasing the levels of cytoplasmic Ca^2+^ during HS, either by increasing the intracellular-extracellular Ca^2+^ gradient or by releasing Ca^2+^ from intracellular stores, improves embryo yield.

There seems to be a relationship between increased Ca^2+^ levels and embryogenic competence, as the inducible (embryogenesis-activatable) microspore stages have higher Ca^2+^ levels than any other microspore/pollen stage, and these levels increase even more as soon as they become induced (this work; Rivas-Sendra et al., 2017, 2019). Since no relevant increases in the percentages of highly embryogenic structures (EE and LBS/SUS) were observed with Ca(NO_3_)_2_ and InsP_3_ (**Figure 5D**), increased embryo yield would come from a Ca^2+^-derived increase of the total number of induced microspores. It is reasonable to speculate that, as any other living population, different microspores may have slightly different endogenous Ca^2+^ levels and/or sensitivities to HS induction, which may make them need different times to become induced. Indirect evidence supporting this notion is the need for 7 days with extra Ca(NO_3_)_2_ to show positive results in a recalcitrant genotype such as DH12075. We hypothesize that keeping Ca^2+^ levels increased with Ca(NO_3_)_2_ or InsP_3_ during 3 days (the inductive period) in DH4079 creates an optimized environment for the “slower” microspores and/or those needing higher Ca^2+^ levels to become induced. In DH12075, a longer exposure would be needed for similar results. This would not be surprising, since this genotype is less sensitive to stress than DH4079 (Camacho-Fernández et al., 2024). Thus, sustainedly high Ca^2+^ levels during the inductive period would increase the number of embryogenic microspores by increasing the amount of microspores reaching the minimum Ca^2+^ level required to be activated.

### Microspore embryogenesis can be modulated by altering Ca^2+^ levels within a functional range

This study and others showed that Ca^2+^ can be considered as an early marker of induction to *in vitro* embryogenesis (Rivas-Sendra et al., 2017, 2019; Calabuig-Serna et al., 2023a, c). Modulation of embryo yield by altering Ca^2+^ levels suggests that Ca^2+^ demands during microspore embryogenesis are dynamic and flexible. Such plasticity, however, has some limits, as revealed by CPA experiments. CPA is a mycotoxin known to affect intracellular Ca^2+^ signaling by inhibiting the ER Ca^2+^ pumps responsible for the return of released Ca^2+^ from the cytoplasm back to the ER (Ostry et al., 2018). We showed that preventing such return with CPA precludes microspore embryogenesis. In line with this, the rupture of the intracellular-extracellular Ca^2+^ gradient with ionophore A23187 was also negative, as also shown for *Arabidopsis* somatic embryogenesis (Calabuig-Serna et al., 2023c) and bread wheat anther culture (Reynolds, 2000). Moreover, the combination of increased intracellular and extracellular Ca^2+^ levels by InsP_3_ and Ca(NO_3_)_2_, respectively, was not beneficial, being even negative for 7-day exposure. Although the possibility of some unspecific detrimental effects of InsP_3_ cannot be ruled out, these results point to an upper threshold above which intracellular Ca^2+^ levels would become toxic.

We used BAPTA-AM and EGTA to chelate intracellular and extracellular Ca^2+^, respectively. We also used W-7 and CPZ as CaM inhibitors whose main physiological effect is to reduce Ca^2+^ signaling without altering Ca^2+^ levels (Liu et al., 2005), and bepridil to block Ca^2+^ channels involved in auxin-mediated Ca^2+^ signaling (De Vriese et al., 2019). The five chemicals produced similar profiles of heavily dose-dependent embryo yield reduction, producing no embryos at the highest concentrations (**Figures 6**, **7**). These results are similar to those found in other *in vitro* embryogenesis systems, including bread wheat microspore embryogenesis and carrot and Arabidopsis somatic embryogenesis (Reynolds, 2000; Calabuig-Serna et al., 2023a, c). These results might reasonably suggest a physiological role for Ca^2+^ in cell signaling upon binding with CaM, considering that Ca^2+^-CaM signaling acts in a wealth of physiological and cellular processes along embryo development (Tian et al., 2020). Thus, despite the plasticity of Ca^2+^ homeostasis, Ca^2+^ levels must be kept between a lower and an upper threshold. As long as Ca^2+^ levels are kept within this range, Ca^2+^ can be used to modulate microspore embryogenesis as it increases the amount of embryogenic microspores.

### High Ca^2+^ levels could be associated to differentiation stages

Freshly isolated microspores and young pollen grains, the stages more sensitive to embryogenesis induction, present Ca^2+^ levels higher than other *in vivo* developmental stages, which led to the notion that Ca^2+^ facilitates embryogenesis induction (Rivas-Sendra et al., 2017, 2019). In this work, induction of microspore embryogenesis is achieved with a 3-day-long HS treatment. Upon HS application, there is a progressive increase of Ca^2+^ levels in induced microspores, but not in non-induced microspores or pollen grains, also exposed to the same HS conditions (**Figures 3**, **4**; Rivas-Sendra et al., 2017). By day 3, the Ca^2+^ levels of growing, induced microspores nearly doubled those of non-growing microspores, and after day 3, once microspores are released from the HS, Ca^2+^ levels kept increasing, peaking at day 5 where Ca^2+^ levels nearly doubled those of freshly isolated microspores. The initial stages (days 1-5, equivalent to days 1-3 of the protocols using 1-day-long HS), are the stages where microspores are induced to embryogenesis and differentiate into embryogenic structures, with no massive cell proliferation. Together, these results suggest a possible role for Ca^2+^ and CaM in the auxin-mediated signaling pathways involved in embryo induction and differentiation. This is supported by the similarities between the profiles of the 3-day, 7-day and continuous exposures to CPZ and bepridil, which suggest that the main effects of these drugs are produced during the first days of culture. However, a redundant effect of CPZ and bepridil cannot be ruled out, as bepridil has also been reported to inhibit CaM in animals (Campbell et al., 1986).

There also was a clearly positive effect for continuous exposures to Ca^2+^-increasing chemicals, which indicates that higher Ca^2+^ availability is also beneficial at embryogenic stages later than day 7, and that these effects are even more positive than for 3-day exposures, as they compensate for the negative effects of 7-day application to produce a net positive result that in some cases (3x Ca(NO_3_)_2_ and 10 µM InsP_3_) is ~80% higher than controls (**Figures 5A, B**). These are the stages when globular embryos change polarity and differentiate into heart-shaped embryos and beyond, when activated CaM shows a polarized distribution (Hause et al., 1994), and when the main differentiation events during embryo development take place (Dresselhaus and Jürgens, 2021). Thus, there seems to be a positive link of Ca^2+^ with embryo differentiation, rather than with undifferentiated cell proliferation.

### The embryogenic microspore as an experimentally Ca^2+^-activatable haploid zygote-like cell

It seems that Ca^2+^ increase (up to a certain limit) promotes the embryogenic development of microspores. This is not surprising considering the scenario of zygotic embryogenesis, where in animal, algal and flowering plant models, Ca^2+^ increase is necessary for egg cell activation and induction of zygote development (Ge et al., 2007; Chen et al., 2015). Following *in vitro* egg cell fertilization, there is an influx of extracellular Ca^2+^ that, in turn, promotes a long-lasting increase of intracellular free Ca^2+^ levels (Digonnet et al., 1997; Antoine et al., 2000). In *in vitro* systems such as cultured microspores and somatic cells, induction of embryogenesis is followed by an increase of intracellular Ca^2+^ levels (Timmers et al., 1989, 1996; Calabuig-Serna et al., 2023a, c). As in sexual zygotes, cultured microspores would react to increases in extracellular Ca^2+^ levels by increasing Ca^2+^ influx. This would explain why increased levels of extracellular and/or intracellular free Ca^2+^ are able to promote microspore embryogenesis, as we hereby showed.

Ca^2+^ peaking during zygotic double fertilization is thought to induce the reorganization of the cytoskeleton and fragmentation of the vacuole needed to establish zygote polarization (Chen et al., 2015). It is to no surprise that cytoskeletal reorganization and fragmentation of the large vacuole of the microspore are among the first cellular changes undergone by embryogenic microspores (Zaki and Dickinson, 1990; Hause et al., 1993). These could well be targets for Ca^2+^-mediated signaling during the first moments of embryogenesis induction. One of the first events shown to occur upon egg cell fertilization is the formation of a cell wall. During maize *in vitro* fertilization studies, the formation of cell wall material was identified as soon as 30 s after gamete fusion (Kranz et al., 1995). Experimenting with Ca^2+^ in unfertilized maize egg cells, Antoine et al. (2000) found that the artificial activation of Ca^2+^ influx was sufficient to produce a new cell wall 40 min after Ca^2+^ influx activation with no need for interaction with sperm cells. This was proposed to be one of the first events of very early embryogenesis initiation, as a mechanism to block polyspermy (Antoine et al., 2000; Chen et al., 2015). In microspores, induction of embryogenesis implies profound cell wall remodeling (Corral-Martínez et al., 2019; Camacho-Fernández et al., 2021), including the formation of a callose-rich, isolating subintinal layer (Parra-Vega et al., 2015b; Rivas-Sendra et al., 2019). At the gene expression level, there are also remarkable coincidences. Soon after fertilization, activation of the zygotic embryo involves the expression of a series of genes from the maternal genome. However, the expressed embryogenesis-promoting transcription factors *BBM, LEC1, WOX2* and *WOX8/9* were found to have a paternal genome origin (Anderson et al., 2017). These factors are also expressed soon upon induction of embryogenesis in microspores (Malik et al., 2007). All these considered, it is reasonable to speculate that the early cellular and molecular changes undergone by induced microspores could well be a reflection of the early changes undergone by activated egg cells. In other words, embryogenic microspores would behave as a sort of experimentally-activatable haploid zygote-like cell.

## Data Availability

The original contributions presented in the study are included in the article/**Supplementary Material**. Further inquiries can be directed to the corresponding author.
