# Supplementary material for: Calcium levels modulate embryo yield in Brassica napus microspore embryogenesis
Source: Front Plant Sci. 2025 Jan 16;15:1512500. doi: 10.3389/fpls.2024.1512500 (PMC11779705; doi:10.3389/fpls.2024.1512500)
Supplement: Supplementary file 1 [file DataSheet1.pdf]

Supplementary materials for:

Calcium modulation is sufficient to modulate embryo yield in *Brassica napus* microspore embryogenesis

Antonio Calabuig-Serna, Ricardo Mir, Daniel Sancho-Oviedo, Paloma Arjona, Jose María Seguí-Simarro*.
* Correspondence: seguisim@btc.upv.es

Supplementary Table S1. Absolute values of the number of embryos produced in each treatment.

$Ca(NO_3)_2$ – DH4079

	3-day exposure				7-day exposure				Continuous exposure			
	Control	2x	3x	4x	Control	2x	3x	4x	Control	2x	3x	4x
Mean	90.33	123.00	132.00	76.00	70.00	73.00	77.00	57.67	47.67	68.67	82.00	54.33
s.e.	4.06	4.04	5.69	3.61	6.24	8.89	2.31	8.65	2.19	3.18	2.08	4.06

$Ca(NO_3)_2$ – DH12075

	3-day exposure				7-day exposure				Continuous exposure			
	Control	2x	3x	4x	Control	2x	3x	4x	Control	2x	3x	4x
Mean	4.00	2.00	0.33	*	4.00	21.67	18.67	6.33	4.00	0.67	0.33	0.33
s.e.	0.00	0.00	0.33	*	0.00	2.03	5.67	1.67	0.00	0.67	0.33	0.33

*: Contaminated

$InsP_3$

	3-day exposure					7-day exposure					Continuous exposure				
	Control	0.1 µM	1 µM	10 µM	100 µM	Control	0.1 µM	1 µM	10 µM	100 µM	Control	0.1 µM	1 µM	10 µM	100 µM
Mean	83.00	98.00	99.67	107.33	79.00	66.67	52.00	71.67	76.33	64.00	47.67	40.00	79.67	87.67	71.33
s.e.	6.24	2.52	3.28	5.36	2.08	4.33	4.16	9.96	2.03	4.36	2.19	2.45	1.45	2.19	4.70

$Ca(NO_3)_2$ + $InsP_3$

	3-day exposure		7-day exposure		Continuous exposure	
	Control	$Ca(NO_3)_2$ + $InsP_3$	Control	$Ca(NO_3)_2$ + $InsP_3$	Control	$Ca(NO_3)_2$ + $InsP_3$
Mean	151.00	131.00	92.33	54.67	50.33	61.33
s.e.	8.72	13.05	4.91	2.40	15.41	6.49

Ionophore A23187

	3-day exposure				Continuous exposure			
	Control	10 µM	20 µM	50 µM	Control	10 µM	20 µM	50 µM
Mean	57.33	22.00	0.00	0.00	74.00	0.00	0.00	0.00
s.e.	13.69	1.53	0.00	0.00	3.46	0.00	0.00	0.00

Cyclopiazonic acid

	3-day exposure					7-day exposure					Continuous exposure				
	Control	0.1 µM	1 µM	10 µM	100 µM	Control	0.1 µM	1 µM	10 µM	100 µM	Control	0.1 µM	1 µM	10 µM	100 µM
Mean	92.00	84.50	45.00	0.00	0.00	88.33	64.75	43.25	0.00	0.00	95.00	88.50	6.50	0.00	0.00
s.e.	2.48	6.76	7.38	0.00	0.00	6.17	2.10	7.32	0.00	0.00	1.73	3.75	1.85	0.00	0.00

BAPTA-AM

	3-day exposure				7-day exposure				Continuous exposure			
	Control	10 µM	20 µM	50 µM	Control	10 µM	20 µM	50 µM	Control	10 µM	20 µM	50 µM
Mean	80.00	73.67	72.33	42.67	31.67	26.00	18.33	8.67	68.67	0.00	0.00	0.00
s.e.	4.04	4.10	3.38	5.36	2.03	2.65	3.18	0.33	3.18	0.00	0.00	0.00

EGTA

	3-day exposure						7-day exposure						Continuous exposure					
	Control	1 µM	10 µM	100 µM	1 mM	10 mM	Control	1 µM	10 µM	100 µM	1 mM	10 mM	Control	1 µM	10 µM	100 µM	1 mM	10 mM
Mean	90.75	74.00	63.50	60.75	0.50	0.00	85.00	76.00	75.67	24.67	0.00	0.00	100.00	77.67	74.00	15.33	0.00	0.00
s.e.	5.12	5.61	7.03	5.25	0.50	0.00	7.23	4.62	2.33	5.78	0.00	0.00	2.65	1.20	4.36	3.18	0.00	0.00

W-7

	3-day exposure			7-day exposure			Continuous exposure		
	Control	50 µM	100 µM	Control	50 µM	100 µM	Control	50 µM	100 µM
Mean	99.33	31.67	2.33	72.00	21.00	0.67	150.67	0.00	0.00
s.e.	3.38	4.98	0.33	1.73	2.08	0.33	28.70	0.00	0.00

Chlorpromazine

	3-day exposure					7-day exposure					Continuous exposure				
	Control	0.1 µM	1 µM	10 µM	100 µM	Control	0.1 µM	1 µM	10 µM	100 µM	Control	0.1 µM	1 µM	10 µM	100 µM
Mean	116.00	114.67	86.33	66.00	1.33	66.33	65.00	32.33	23.00	0.00	122.33	103.67	103.00	24.33	0.00
s.e.	7.21	3.18	5.93	7.02	0.88	2.60	5.29	5.04	2.65	0.00	8.97	3.28	1.15	3.84	0.00

Bepridil

	3-day exposure						7-day exposure						Continuous exposure					
	Control	5 µM	10 µM	25 µM	50 µM	100 µM	Control	5 µM	10 µM	25 µM	50 µM	100 µM	Control	5 µM	10 µM	25 µM	50 µM	100 µM
Mean	91.50	97.50	84.25	51.25	0.00	0.00	87.75	76.50	56.25	37.75	0.00	0.00	80.50	79.67	75.25	45.75	0.00	0.00
s.e.	4.84	6.06	5.19	3.09	0.00	0.00	5.57	7.41	6.42	5.14	0.00	0.00	3.23	3.93	6.34	6.10	0.00	0.00

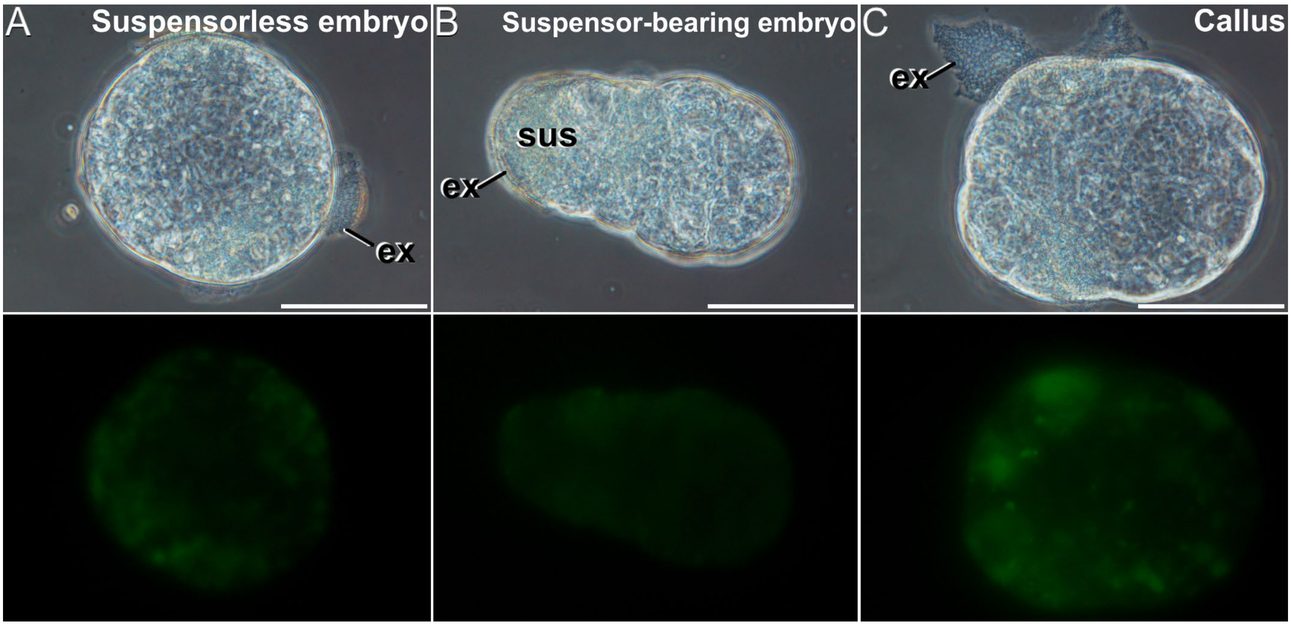

Suppl. Fig. S1. Ca^{2+} detection with FluoForte in 8-day-old *B. napus* microspore cultures. Paired images of the same microscopic field imaged by phase contrast optics (top image) and fluorescence (bottom image). A: Suspensorless globular embryo. B: Suspensor-bearing globular embryo. C: Callus. ex: exine; sus: suspensor. Bars: 50 µm.

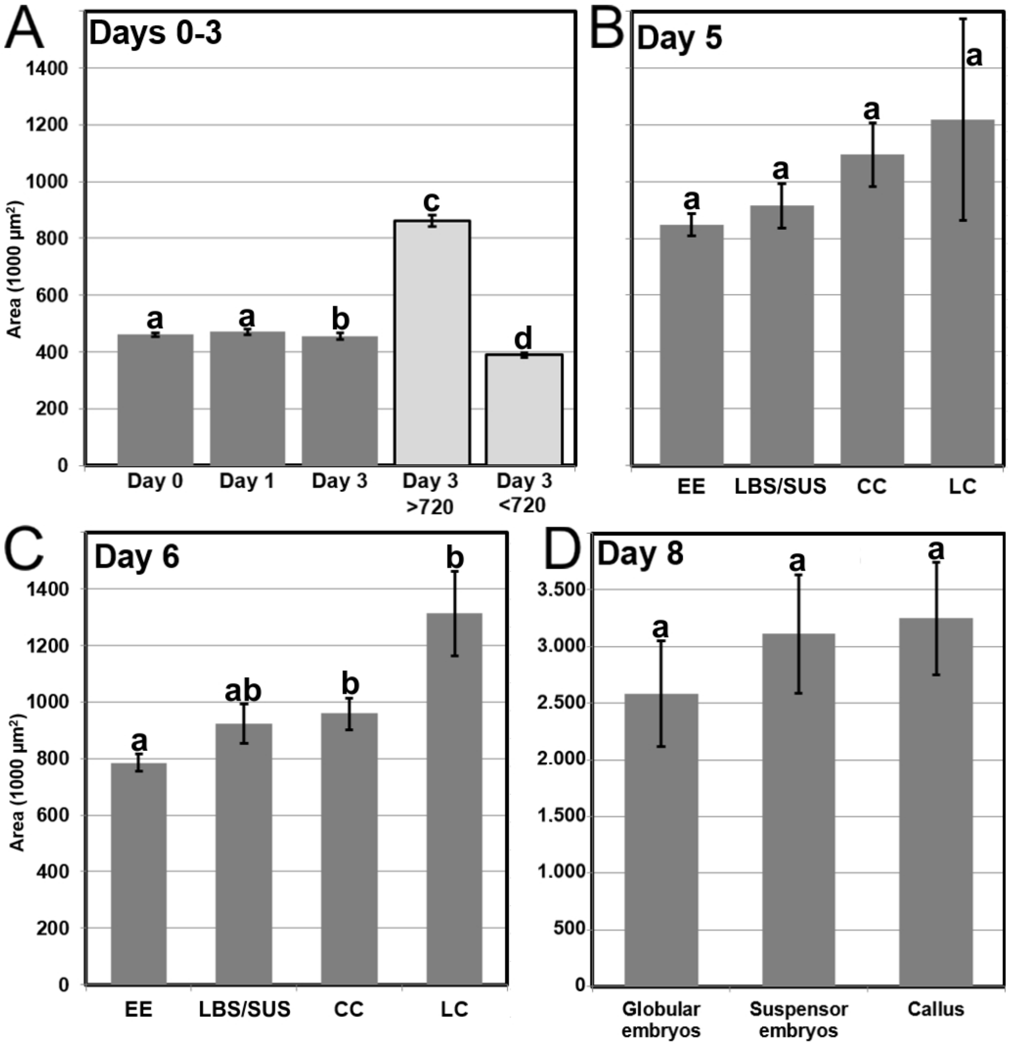

Suppl. Fig. S2. **Quantification of the average area of the different structures developed in *B. napus* microspore cultures**. Average area is expressed in 1000 µm^2 ± standard error (error bars). A: Average areas in microspores of day 1, day 2 and day 3 cultures. Light grey bars represent a segregation of total day 3 structures in two categories: structures smaller and larger than 720 µm^2 (see text for further details). B, C: Average area of the different embryogenic structures (EE, LBS/SUS, CC and LC) identified in five (B) and six-day-old cultures (C). D: Average area of the different suspensorless globular embryos, suspensor-bearing embryos and calli observed in eight-day-old cultures. All areas were measured under identical experimental conditions and are represented using the same scale, except for day 8 structures. For each chart, different letters indicate significant differences according to the Kruskal-Wallis test ($p \leq 0.05$).

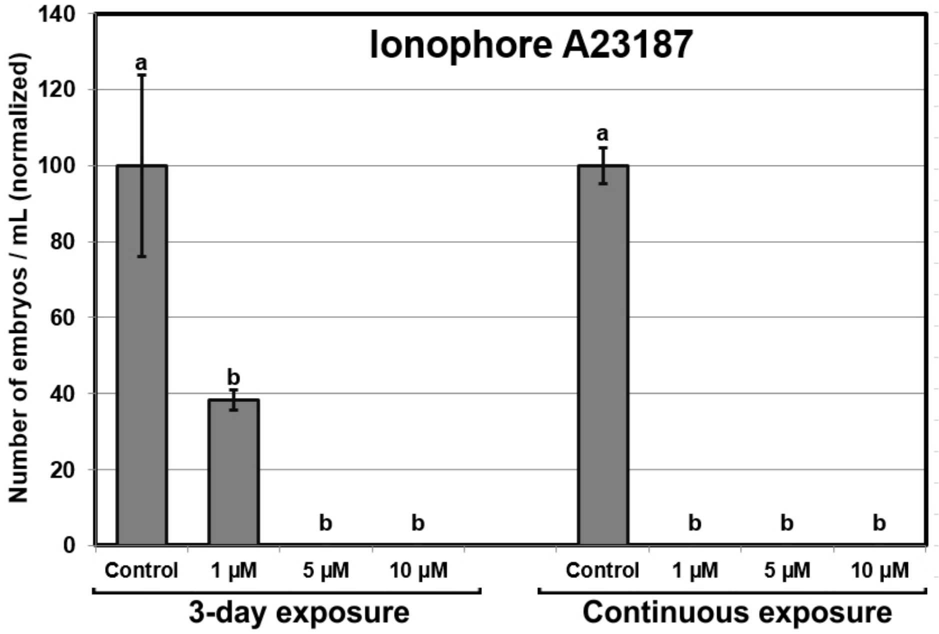

Suppl. Fig. S3. Effects of adding ionophore A23187. Ionophore A23187 was added to the culture medium at 0 (control) 1, 5 and 10 µM and during the first three days of culture and continuously. Effects are expressed as number of embryos produced per mL of culture medium, normalizing control values to 100. Different letters indicate significant differences according to the LSD test (p≤0.05).

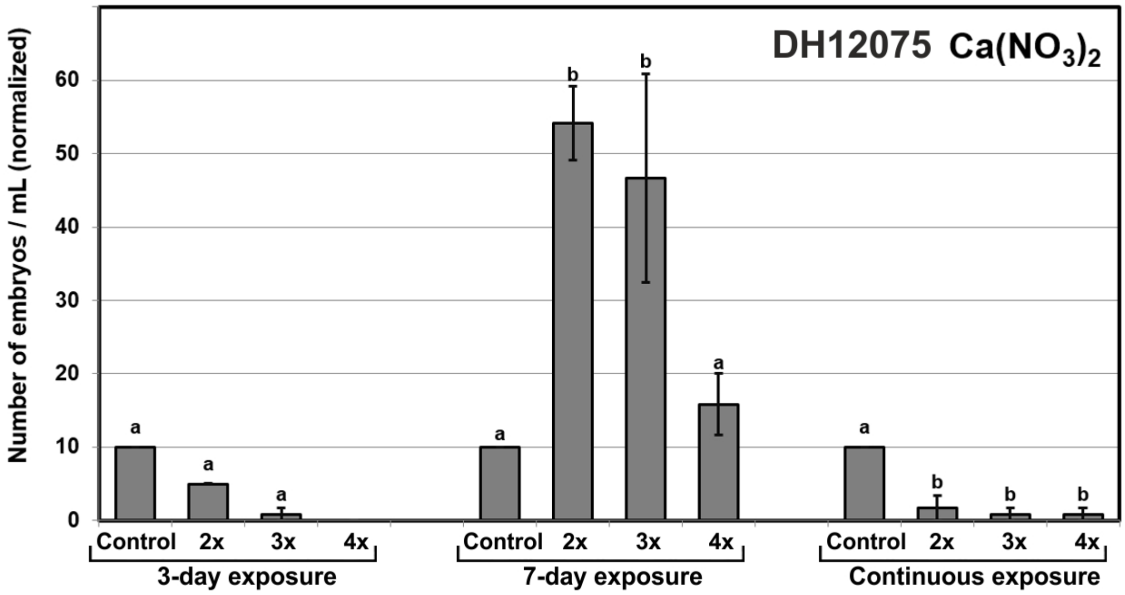

Suppl. Fig. S4. Effects of increasing Ca^{2+} availability in the DH12075 line with the addition to the culture medium of different concentrations of $Ca(NO_3)_2$. The different $Ca(NO_3)_2$ concentrations were applied during the first three days of culture, during seven days, and continuously. Effects are expressed as number of embryos produced per mL of culture medium, normalizing control values to 10 embryos/mL. Different letters indicate significant differences according to the LSD test ($p \leq 0.05$).
